# Enhanced Anti-diabetic Effect of Berberine Combined With Timosaponin B2 in Goto-Kakizaki Rats, Associated With Increased Variety and Exposure of Effective Substances Through Intestinal Absorption

**DOI:** 10.3389/fphar.2019.00019

**Published:** 2019-01-24

**Authors:** Xiaoting Tian, Fang Liu, Zhixiong Li, Yunfei Lin, Huan Liu, Pei Hu, Mingcang Chen, Zhaolin Sun, Zhou Xu, Yiting Zhang, Li Han, Yuanyuan Zhang, Guoyu Pan, Chenggang Huang

**Affiliations:** ^1^Shanghai Institute of Materia Medica, Chinese Academy of Sciences, Shanghai, China; ^2^University of Chinese Academy of Sciences, Beijing, China; ^3^West China School of Basic Medical Sciences and Forensic Medicine, Sichuan University, Chengdu, China

**Keywords:** berberine, timosaponin B2, diabetes, combination treatments, drug–drug interaction, metabolites, hepatic metabolism, intestinal absorption

## Abstract

**Objective:** Inspired by the traditionally clinical application of herb pair *Zhimu*-*Huangbo* to treat diabetes, a combination of plant ingredients, timosaponin B2 (TB-2) and berberine (BBR), was evaluated for their anti-diabetic efficacy and cooperative mechanisms.

**Methods:** The efficacy and pharmacokinetics of orally administered TB-2 (33.3 mg/kg/day), BBR (66.7 mg/kg/day), and TB-2+BBR (100 mg/kg/day) were evaluated in spontaneously non-obese diabetic Goto-Kakizaki (GK) rats, and metformin (200 mg/kg/day) was used as a positive control. The comparative exposure of the parent drugs, timosaponin A3 (TB-2 metabolite), and M1–M5 (BBR metabolites) was quantified in the portal vein plasma (before hepatic disposition), liver, and systemic plasma (after hepatic disposition) of normal rats on single and combination treatments. Cooperative mechanism of TB-2 and BBR on intestinal absorption and hepatic metabolism was investigated in Caco-2 cells and primary hepatocytes, respectively.

**Results:** After a 6-week experiment, non-fasting and fasting blood glucose levels and oral glucose tolerance test results showed that TB-2+BBR treatments (100 mg/kg/day) displayed significantly anti-diabetic efficacy in GK rats, comparable to that on metformin treatments. However, no significant improvement was observed on TB-2 or BBR treatments alone. Compared to single treatments, combination treatments led to the increased circulating levels of BBR by 107% in GK rats. In normal rats, the hepatic exposure of BBR, timosaponin A3, and M1–M5 was several hundred folds higher than their circulating levels. Co-administration also improved the levels in the plasma and liver by 41–114% for BBR, 141–230% for TB-2, and 12–282% for M1–M5. *In vitro*, the interaction between TB-2 and BBR was mediated by intestinal absorption, rather than hepatic metabolism.

**Conclusion:** Combining TB-2 and BBR enhanced the anti-diabetic efficacy by increasing the *in vivo* variety of effective substances, including the parent compounds and active metabolites, and improving the levels of those substances through intestinal absorption. This study is a new attempt to assess the effects of combined plant ingredients on diabetes by scientifically utilizing clinical experience of an herb pair.

## Introduction

Type 2 diabetes (T2D) mellitus, a chronic, complex metabolic disorder with serious and lethal complications, is becoming increasingly common throughout the world. The prevalence of diabetes has been predicted to give rise to 592 million cases by 2035 (Guariguata et al., 2014). Although several oral synthetic anti-diabetic drugs, such as metformin and sulfonylureas, are available, patients taking these medications continue to suffer from poor long-term efficacy and high complication rates (Nathan, 2007; Nathan et al., 2009). Due to the advantages of fewer side effects and positive perceptions of patients, herbal medicines or their active natural ingredients are increasingly popular around the world, especially for chronic metabolic diseases. In ancient China, T2D was highly consistent with “*Xiao ke*,” a symptom characterized as “three excesses and one loss,” including excessive fluid intake, food consumption, and urination, along with weight loss (Wang, 2003). “*Xiao ke*” was first described in “*Effective Prescriptions of the Past and Present*” written during the Tang Dynasty (AD 600), nearly one century earlier than the description of diabetes by Western people in 1674 (Wang, 2003). Through their long history of clinical usage, herbal medicines, predominantly in the form of combinations of herbal medicines, have shown excellent therapeutic efficacy. *Zhimu*-*Huangbo*, composed of the dried rhizome of *Anemarrhena asphodeloides Bunge* (*Zhimu* in Chinese) and dried bark of *Cortex Phellodendri Chinensis* (*Huangbo* in Chinese), is one of the famous herb pairs for treating “*Xiao Ke*,” which was originally recorded in “*Lan shi mi cang*” written during the Jin Dynasty (AD 300). Currently, some relevant commercial products, such as *Zishen* *Wan* and *Zhibai Dihuang Pill*, are available in the market for treating diabetes or relevant symptoms. Correspondingly, our team firstly confirmed the anti-diabetic effect of *Zishen Wan* in spontaneously T2D mice (Tang et al., 2012). Zhao et al. (2012) demonstrated that *Zhibai Dihuang* *Pill* ameliorated diabetic symptoms in streptozotocin-induced diabetic rats. Other teams also confirmed the anti-diabetic efficacy of *Zhimu-Huangbo* in diabetic animals (Zhang et al., 2014; Song et al., 2015).

Compared with herbal medicines with complicated compositions and unclear scientific standards, evidence-based active natural ingredients are more likely to be understood and accepted. Due to the unsatisfactory therapeutic effects of monotherapy, combination therapy has become a preferable choice for diabetic patients (Prabhakar et al., 2014). Moreover, the low bioavailability of most natural ingredients calls for improved levels in target organs, and drug–drug interactions (DDIs) may be employed as an applicable strategy for solving this problem (Liu et al., 2016). The combination of several herbs can serve as a good template for developing combination treatments of natural ingredients for diabetes. Thus, our team has made many efforts over years to elucidate the medical material basis of *Zhimu-Huangbo* on diabetic treatments, companied by some published papers regarding this herb pair, single herbs, and active ingredients (Ma et al., 2008, 2009; Tian et al., 2014, 2016a,b,c; Fu et al., 2015; Jia et al., 2015; Sheng et al., 2015). Finally, after many pharmacodynamic comparisons, berberine (BBR) in *Huangbo* and timosaponin B2 (TB-2) in *Zhimu* were selected for combination therapy for diabetes.

**FIGURE 1:**
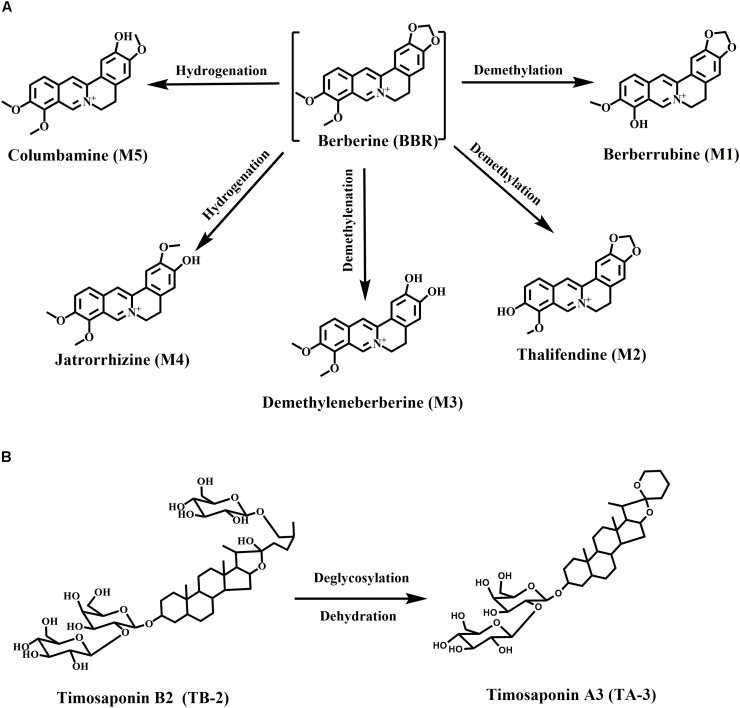
Chemical structures and metabolic pathways of **(A)** BBR and **(B)** TB-2.

Berberine, a well-known isoquinoline alkaloid and an over-the-counter drug for gastrointestinal infection, has received considerable renewed attention due to its anti-diabetic potential via multiple proposed pathways (Yao et al., 2015). Recently, various researchers have confirmed the blood glucose-lowering activities of BBR in patients and diabetic animals, along with its great efficacy against diabetes-induced complications (Zhang and Chen, 2012). However, the major drawback of BBR is the low bioavailability (usually <1% in various species) (Liu et al., 2016), especially considering that high oral doses of BBR (>900 mg/day) carry a risk of clinical gastrointestinal side effects (Yin et al., 2008). Moreover, the major BBR metabolites, M1–M5 (Figure 1A), were also verified to have hypoglycemic activity in cells or animals (Li et al., 2011; Wang K. et al., 2017; Yang et al., 2017). M1 even showed more hypoglycemic capacity than BBR in diabetic animals (Yang et al., 2017).

Timosaponin B2 (TB-2), the major bioactive steroid saponin with the highest content in *Zhimu*, has been a focus of our team for many years. Previously, we found that BBR and TB-2 were both the substrates of uptake transporter organic anion-transporting polypeptide (OATP) (Chen et al., 2015; Sheng et al., 2015). Moreover, we clarified the metabolism, hepatic disposition, and DDIs of TB-2 (Fu et al., 2015; Jia et al., 2015; Sheng et al., 2015; Tian et al., 2016b). As reported, TB-2 significantly decreased blood glucose levels and ameliorated diabetic nephropathy *in vivo*, and it improved insulin sensitivity *in vitro* (Yuan et al., 2015, 2016). Timosaponin A3 (TA-3, Figure 1B), a major metabolite of TB-2 previously confirmed by our team (Fu et al., 2015; Jia et al., 2015), was found to lower blood glucose levels in diabetic rats and mice, probably through inhibition of hepatic gluconeogenesis or glycogenolysis (Nakashima et al., 1993). Of note, in addition to being the predominant metabolic organ, liver is also the major target organ for treating diabetes with the capacity to produce glucose. Correspondingly, various anti-diabetic pathways of BBR, TB-2, and their metabolites have been clarified, such as adenosine 5′-monophosphate protein kinase (AMPK) activation, insulin receptor (InsR) expression improvement, and liver gluconeogenesis inhibition (Zhang et al., 2008; Dong et al., 2012; Pirillo and Catapano, 2015).

Goto-Kakizaki (GK) rats, a spontaneously non-obese T2D animal model, were created through selectively breeding Wistar rats according to glucose intolerance over multiple generations (Akash et al., 2013). In this study, we compared the anti-diabetic efficacy and PK patterns of single (BBR at 66.7 mg/kg/day or TB-2 at 33.3 mg/kg/day) and combined (100 mg/kg/day) treatments in GK rats. To elucidate the anti-diabetic material basis, the quantitative analysis of BBR, TB-2, and their effective metabolites (Figure 1) was systematically performed from a liver-centric perspective, namely, in the portal vein plasma(before hepatic clearance), liver, and systemic plasma (after hepatic clearance), following oral intake of BBR (66.7 mg/kg), TB-2 (33.3 mg/kg), and their combination (100 mg/kg) in normal rats. Finally, to preliminarily explore the cooperative mechanisms of BBR and TB-2, Caco-2 cells and primary rat hepatocytes were employed to evaluate the DDIs in intestinal absorption and hepatic metabolism *in vitro*, respectively, which are two major influencing factors on the *in vivo* disposition of orally administrated compounds.

## Materials and Methods

### Materials and Reagents

Timosaponin B2 (>98% pure) for oral administration and reference was isolated from a *Rhizoma Anemarrhenae* decoction by our laboratory. BBR for oral administration (>97% pure) and reference (>98% pure) was purchased from Chengdu Longquan Hi-Tech Natural Pharmaceutical Co., Ltd. (Chengdu, China) and the National Institute for Control of Pharmaceutical and Biological Products Co., Ltd. (Beijing, China), respectively. Thalifendine (M2) (>98% pure) was synthesized in our laboratory. Berberrubine (M1), demethyleneberberine (M3), jatrorrhizine (M4), columbamine (M5), and tolbutamide (internal standard, IS) were purchased from Chengdu Must Bio-Technology Co., Ltd. (Chengdu, China, >98% pure). Acetonitrile and formic acid were purchased from Merck (Darmstadt, Germany). Triple-deionized water was prepared with a Milli-Q system (Millipore, United States). Dimethyl sulfoxide (DMSO), Dulbecco’s modified Eagle’s medium (DMEM), 2-(*N*-morpholino) ethanesulfonic acid (MES), fetal bovine serum, and Hanks’ balanced salt solution (HBSS) were purchased from Sigma–Aldrich Co., Ltd. (St. Louis, MO, United States). All other analytical-grade agents were obtained from the Sino Pharm Chemical Reagent Co., Ltd. (Shanghai, China).

### Animals

Male GK rats, normal age-matched Wistar rats, and high-fat diet were provided by Shanghai SLAC Laboratory Animal Co., Ltd. (Shanghai, China). All the animals were maintained in air-conditioned animal quarters under controlled temperature (23 ± 1°C), noise (<50 db), and relative humidity (60 ± 10%), with a 12 h light/dark cycle (6:00–18:00). The rats were given *ad libitum* access to standard diet and water.

### Instrumentation

Analyses were performed on a Shimadzu LCMS-8030 triple quadrupole system (Shimadzu Corp., Japan) equipped with an electrospray ionization source. Data processing was performed using Shimadzu LC–MS LabSolutions (version 5.42 SP4, Shimadzu, Japan). Chromatographic separation was carried out on a Waters CORTECS C18+ column (100 mm × 2.1 mm, 2.7 μm, Ireland) with a security guard C18+ column (5 mm × 2.1 mm, 2.7 μm, Ireland). The analysis of BBR and M1–M5 was optimized as follows: the mobile phase consisted of acetonitrile containing 0.2% formic acid (A) and water containing 0.2% formic acid and 2.5 mM of ammonium acetate (B), and the flow rate was set to 0.2 ml/min with a 10-μl injection volume. The gradient elution was 85% B from 0 to 2.5 min, 85% B–83% B from 2.5 to 3.0 min, 83% B from 3.0 to 6.0 min, 83% B–40% B from 6.0 to 6.5 min, 40% B from 6.5 to 7.0 min, 40% B–85% B from 7.0 to 7.2 min, and 85% B from 7.2 to 12 min. The optimized precursor-to-product ion pairs (collision energy) were in the positive mode as follows: *m*/*z* 336.00–320.10 (22 V) for BBR (at 8.9 min), 322.20–307.10 (22 V) for M1 (at 8.7 min) and M2 (at 6.8 min), 324.10–308.10 (24 V) for M3 (at 4.20 min), 338.2–323.10 (24 V) for M4 (at 7.3 min) and M5 (at 6.8 min), and 271.50–154.70 (25 V) for tolbutamide (IS, at 9.80 min). The analysis of TB-2 and its metabolite (TA-3) was optimized as follows: the mobile phase consisted of acetonitrile (A) and water containing 0.1% formic acid (B), and the flow rate was set to 0.2 ml/min with a 10-μl injection volume. The gradient elution was 88% B–70% B from 0 to 0.1 min, 70% B from 0.1 to 2.0 min, 70% B–10% B from 2.0 to 3.0 min, 10% B–2% B from 3.0 to 3.5 min, 2% B from 3.5 to 6.8 min, 2% B–88% B from 6.8 to 7.0 min, and 88% B from 7.0 to 11.0 min. The optimized precursor-to-product ion pairs (collision energy) were in the positive mode as follows: *m*/*z* 579.20–255.15 (28 V) for TB-2 (at 4.1 min) and timosaponin A3 (at 6.1 min) and 271.50–154.70 (25 V) for tolbutamide (IS, at 5.8 min). The calibration standards were prepared by spiking the standard solutions in blank plasma to achieve the final concentrations of 1–500 ng/ml for TB-2 and TA-3, as well as 0.5–250 ng/ml for BBR and its metabolites (M1–M5). The calibration standards were prepared by spiking the standard solutions into the liver homogenates to yield final concentrations of 3–3000 ng/ml for TA-3, and 1–1000 ng/ml for TB-2, BBR, and M1–M5. If the concentrations of the compounds were over the linear range, the relevant samples were diluted with blank matrix by a factor of 10. All the test compounds showed good linearity with *R*^2^ > 0.99, and more than 66.7% of the control samples had a target accuracy within ±20% standard deviation (SD).

### Anti-diabetic Efficacy and PK in GK Rats

Four-week-old male GK rats were fed a high-fat diet, and age-matched non-diabetic Wistar rats were fed standard chow as the control group. Under these dietary conditions, blood glucose was estimated each week for 7 weeks in 12-h fasted rats using a portable glucometer (One Touch Sure Step Meter, Johnson & Johnson, United States) by drawing the blood from the tail vein. GK rats with fasting blood glucose levels (FBG) above 16.7 mM were considered as diabetic rats, and only uniformly diabetic rats were used in this study. At 11 weeks of age, the GK rats were randomly divided into five groups (*n* = 8): the vehicle group, TB-2 group (33.3 mg/kg/day), BBR group (66.7 mg/kg/day), TB-2+BBR (33.3+66.7 mg/kg/day) group, and metformin group (200 mg/kg/day) as a positive control. All the drugs were suspended in 0.5% carboxymethylcellulose sodium (CMC-Na) solution for oral gavage (10 ml/kg) once per day (at 9:00) for 6 weeks. Simultaneously, the Wistar and vehicle groups received vehicle over the treatment period.

Food consumption and body weight were measured every week throughout the experimental periods. Non-fasting blood glucose (NFBG) levels were determined by collecting blood samples from the tail vein (at 9:00), and FBG levels were determined after a 6-h fast (at 15:00) on days 0, 7, 14, 21, and 42. On day 28, an oral glucose tolerance test (OGTT) was performed after an overnight fast. After the rats received oral administration of glucose solution (2.0 g/kg), the blood glucose levels were measured at 0, 30, 60, 90, and 120 min after glucose administration. On day 35, after different treatments, approximately 250 μl of blood was collected from the tail vein of the GK rats in each group at 0.5, 1, 2, 4, 6, 12, and 24 h. The blood samples were centrifuged at 10,000 rpm for 5 min, and all the samples were stored at −20°C until analysis. On day 42, the rats were anesthetized with urethane (1.4 g/kg, dissolved in saline) after an overnight fast and were sacrificed to collect the liver, kidney, heart, and spleen. The organs were weighed after washing with saline and drying to calculate the organ coefficient, namely, the ratio between the weight of organs and the body in each rat.

### PK and Liver Distribution in Wistar Rats

Male Wistar rats (135; 250 ± 20 g) were acclimated for 7 days before use. The rats were given *ad libitum* access to standard diet and water. They were randomly divided into three groups (45 animals per group) and fasted overnight with free access to water before treatment. An oral dose of TB-2 (33.3 mg/kg) was administered to the rats in group A. BBR (66.7 mg/kg) was administered to the rats in group B through oral gavage. A combination of TB-2 (33.3 mg/kg) and BBR (66.7 mg/kg) was orally administered to the rats in group C. Forty-five rats per group (*n* = 5 for each time point) were anesthetized with urethane (1.4 g/kg) and sacrificed to collect hepatic portal vein blood (2 ml), abdominal aorta blood (6–8 ml), and liver (2 g) at 0.167, 0.5, 1, 2, 4, 6, 9, 12, and 24 h after oral administration. The blood samples were centrifuged at 10,000 rpm for 5 min for getting the plasma, and the liver samples were washed with saline three times. All the samples were stored at −20°C until analysis.

### Hepatic Metabolism in Primary Hepatocytes

The influence of hepatic metabolism on the DDIs of TB-2 and BBR was investigated using freshly isolated primary rat hepatocytes. Hepatocytes were isolated from male Wistar rats via two-step perfusion, as previously described (Shen et al., 2012; Sheng et al., 2015). The hepatocytes were incubated in six-well plates at a density of 0.5 × 10^6^/well, followed by the addition of TB-2 (1 μM) in the absence or presence of BBR (5, 50 μM), and the addition of BBR (1 μM) in the absence or presence of the CYP enzyme inhibitor 1-aminobenzotriazole (ABT) (1 mM) or TB-2 (0.1, 0.5, 5, and 30 μM). The cells were incubated on an orbital shaker at 37°C for 3 h. Cell suspensions (300 μl) were collected at 0 and 3 h and were immediately mixed with ice-cold methanol (300 μl) to terminate any potential reactions. The cell samples were then lysed by sonication, and each sample (100 μl) was extracted via acetonitrile precipitation, and prepared for analysis by HPLC–MS/MS.

### Transport in Caco-2 Cells

Caco-2 cells at passage 39 were seeded onto Millipore Transwell^®^ 24-well culture plate inserts (Corning 0.4-μm pore polycarbonate membrane, Sterile Co., Bedford, MA, United States) at a density of 1.5 × 10^5^ cells/cm^2^. The culture medium was changed every other day for the first 7 days and then daily for the next 14 days. On day 21 of culture, the integrity of the cell monolayers was evaluated via transepithelial electrical resistance (TEER) measurements using a Millicell-ERS epithelial voltohmmeter (Millipore Co., Ltd.), with required TEER values of 300–1000 Ω/cm for the subsequent transport experiments. Prior to the transport experiments, the cell monolayers were washed three times with HBSS at 37°C. HBSS–MES transport medium (pH 6.0, containing 10 mM of MES) and 1.5 ml of HBSS–HEPES transport medium (pH 7.4, containing 10 mM of HEPES) with or without TB-2 (5 μM), BBR (10 μM), or TB-2 (5 μM)+BBR (10 μM) were added to the apical and basolateral sides. At the same time, the corresponding opposite side was filled with medium as the receiving chamber. After incubation for 30, 60, 90, and 120 min, 250-μl aliquots of the medium was taken from the receiving chamber at each time point, and prepared for analysis by HPLC–MS/MS.

### Sample Preparation

The liver samples were diluted with 3 volumes (0.5 g/1.5 ml) of saline and homogenized. The liver homogenates and plasma samples (100 μl) were precipitated with three volumes of acetonitrile (containing 10 μg/ml tolbutamide). The samples were then vortexed for 5 min and centrifuged at 13,000 rpm for 10 min, after which the supernatant (250 μl) was removed, and the samples were evaporated to dryness under a vacuum at 40°C. Then, 100 μl of the original mobile phase was added, and the samples were vortexed for 5 min and centrifuged at 13,000 rpm at 4°C for 10 min. Finally, the supernatant was used for HPLC–QQQ–MS/MS analysis.

### Data Analysis

A non-compartmental analysis was performed using WinNonlin software (Pharsight 6.2, Raleigh, NC, United States) to calculate the PK parameters. The significance of PK parameters in normal rats, generated by the sparse method in WinNonlin, was assessed with Welch’s unequal variances *t*-test. The significance of the other results was assessed using two-tailed Student’s *t*-tests. Statistical significance was defined as *P* < 0.05.

The liver extraction ratio (ER_liver_) indicates the fraction of hepatic clearance from the liver portal vein plasma to the systemic plasma, and it was calculated as follows:

(1) $${\text{ER}}_{\text{liver}}\;=\;({\text{AUC}}_{\text{por}}-{\text{AUC}}_{\text{sys}})/{\text{AUC}}_{\text{por}}$$

where AUC_por_ and AUC_sys_ are the areas under the concentration–time curve calculated in the portal vein plasma and systemic plasma, respectively.

The apparent permeability (*P*_app_) of drugs across the Caco-2 cell monolayers was calculated from the linear portion (i.e., 30, 60, 90, and 120 min) of a plot of the total amount of drug transported versus time, as follows:

(2) $$P_{\text{app}}=(\Delta Q/\Delta t)/(A*C0)$$

where (Δ*Q*/Δ*t*) is the linear slope of the drug concentration in the receptor chamber over time, *A* represents the surface area of the membrane (1.12 cm^2^), and *C*0 is the initial concentration of drug in the donor chamber.

The efflux ratio (ER_permeability_) for BL to AP and AP to BL transport was defined by the following equation:

(3) $${\text{ER}}_{\text{permeability}}\;=P_{\text{app}}\;(\text{BL}-\text{AP})/P_{\text{app}}\;(\text{AP}-\text{BL})$$

where *P*_app_ (BL–AP) and *P*_app_ (AP–BL) are the apparent permeability of drugs from the basolateral (BL) to apical (AP) and AP to BL sides of the monolayer, respectively.

## Results

### Body Weight, Food Intake, and Organ Coefficients in GK Rats

**FIGURE 2:**
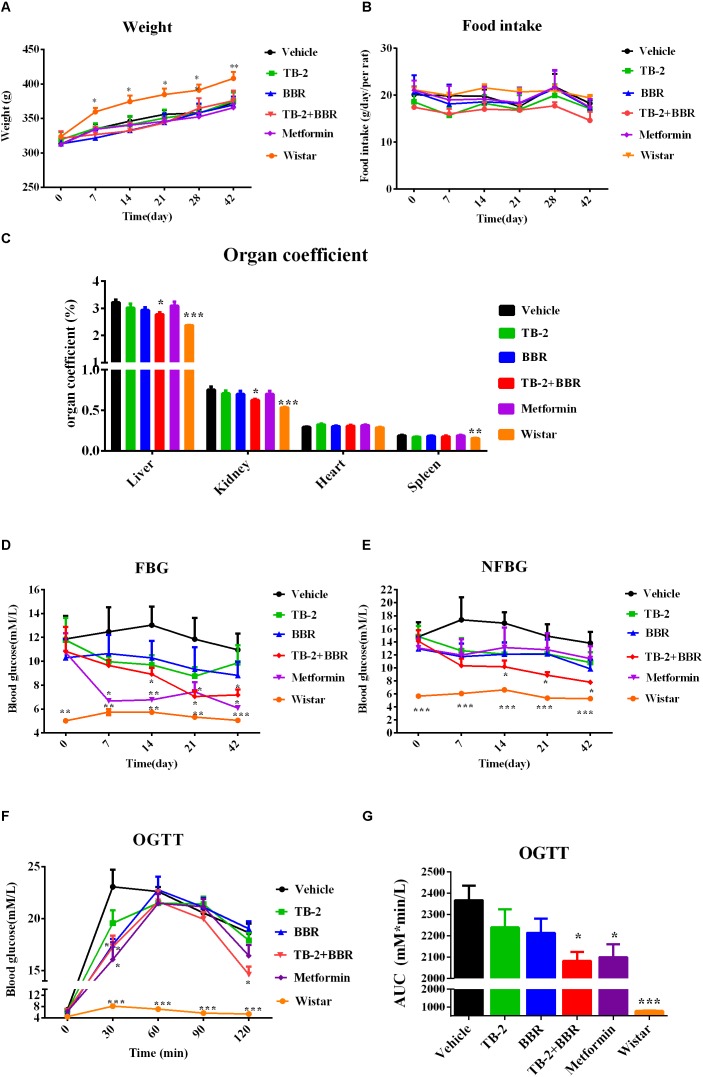
Hyperglycemic parameters **(A)** weight, **(B)** food intake, **(C)** organ coefficient, **(D)** FBG levels, and **(E)** NFBG levels on the vehicle, TB-2 (33.3 mg/kg/day), BBR (66.7 mg/kg/day), TB-2+BBR (100 mg/kg/day), and metformin (200 mg/kg/day) treatments for 6 weeks in GK rats; Wistar rats were used as the control group. **(F)** The levels and **(G)** AUC of the blood glucose during OGTT test in GK rats (*n* = 8). ^∗^*P* < 0.05, ^∗∗^*P* < 0.01, and ^∗∗∗^*P* < 0.001 compared with the vehicle group.

As shown in Figures 2A,B, compared with the age-matched Wistar rats, the GK rats had lower body weights (*P* < 0.0 or *P* < 0.01) but similar food intake (*P* > 0.05) during the experimental period, consistent with published data (Jeong et al., 2009). Compared with the vehicle group, there were no significant differences in the body weight or food intake (*P* > 0.05) in any drug-treated groups, suggesting that orally administered TB-2 (33.3 mg/kg) and BBR (66.7 mg/kg) had no effect on the body weight or food consumption of GK rats receiving single or combined treatments.

As presented in Figure 2C, the liver, kidney, and spleen coefficients were significantly different (*P* < 0.001 or *P* < 0.01) between the vehicle and Wistar groups, indicating that diabetes significantly increased the organ coefficients of the GK rats. The positive control, metformin, had no effect on the decreased organ coefficients compared with the vehicle group. However, a significant decrease in the liver and kidney coefficients (*P* < 0.05) was observed on TB-2+BBR treatments, while this was limited on TB-2 or BBR treatments alone. These results indicated that the combination of TB-2 and BBR improved the physiological state of liver and kidney induced by diabetes in rats.

### NFBG and FBG in GK Rats

As presented in Figures 2D,E, the measured FBG levels were much higher in the GK rats than those in the age-matched Wistar rats on days 0, 7, 14, 21, and 42, as were the NFBG levels, thereby validating the stability of diabetes for pharmaceutical testing in GK rats. Despite slightly reduced trends in the FBG and NFBG levels, there were no significant differences (*P* > 0.05) during the 6-week experiment on either TB-2 or BBR treatments alone compared with the vehicle group. In contrast, the combination of TB-2 and BBR not only caused a greater decrease in FBG and NFBG levels than any single compound, but also exhibited a significant difference (*P* < 0.05) from day 14 to the end of the experiment, compared with the vehicle group. The metformin treatments also significantly decreased FBG levels from the first to the sixth week (*P* < 0.05 or *P* < 0.01). Nevertheless, metformin did not significantly decrease NFBG levels (*P* > 0.05) throughout the whole experiment. At the end of the experiment, compared with the vehicle group, oral administration of TB-2 or BBR alone didn’t significantly decrease FBG or NFBG levels (*P* > 0.05), while co-administration of TB-2 and BBR decreased FBG and NFBG levels by 44.4% and 43.4% with statistical significance (*P* < 0.05), respectively, comparable to the effect on metformin treatments.

### OGTT in GK Rats

After 4 weeks of treatment, an OGTT was performed with oral administration of glucose at 2 g/kg in GK rats, as shown in Figure 2F. Despite the similar levels at the zero time point, the blood glucose levels in GK rats were much higher than those in Wistar rats (*P* < 0.001) after glucose injection. Unlike the blood glucose levels in the vehicle and Wistar rat groups, which peaked at 30 min, the peak values in the drug-treated groups occurred at 60 min. Compared with the vehicle group, the co-administration of TB-2 and BBR significantly decreased the blood glucose levels by 22% (*P* < 0.05) and 25% (*P* < 0.05) at 30 and 120 min, respectively, while the significant decreases of 30% (*P* < 0.05) and 24% (*P* < 0.05) occurred only at 30 min on the metformin and BBR treatments, respectively. To further compare the anti-diabetic efficacy, the area under the time–blood glucose curve (AUC) was calculated. As exhibited in Figure 2G, compared with the vehicle group, the AUCs of blood glucose didn’t significantly decrease (*P* > 0.05) in the GK rats treated with TB-2 or BBR alone. In contrast, the combination of TB-2 and BBR led to a significant decrease of 12.0% in the AUC of blood glucose (*P* < 0.05), which was slightly better than the decrease (11.3%, *P* < 0.05) on metformin treatments.

### PK in GK Rats

**FIGURE 3:**
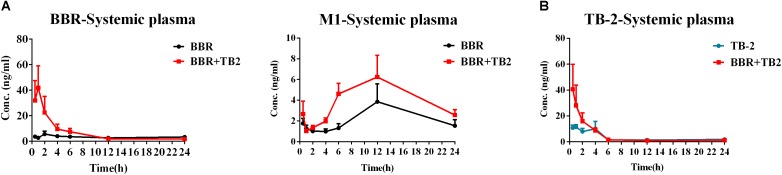
Time–concentration curves of parent compounds and metabolites in the systemic plasma of GK rats after repeated oral administration of **(A)** BBR (66.7 mg/kg/day) vs. TB-2+BBR (33.3+66.7 mg/kg/day) and **(B)** TB-2 (33.3 mg/kg/day) vs. TB-2+BBR (33.3+66.7 mg/kg/day) for 5 weeks (*n* = 8).

After 5 weeks of repeated treatments, a comparative PK evaluation of TB-2, BBR, and the relevant metabolites was conducted in GK rats treated with orally administered TB-2 (33.3 mg/kg), BBR (66.7 mg/kg), and TB-2+BBR (33.3+66.7 mg/kg), as shown in Figure 3 and Table 1. BBR and M1, a major metabolite, were observed in the systemic plasma after oral administration of BBR and TB-2+BBR in GK rats. Compared with BBR treatments alone, the combination treatments led to obvious increases in the *C*_max_ and AUC of BBR by 695% (*P* < 0.05) and 107% (*P* < 0.05), respectively, along with shortened *T*_max_ and *T*_1/2_, while it didn’t significantly increase the AUC or *C*_max_ of M1 (*P* > 0.05). The levels of M1 accounted for 75% and 61% of the parent drug in BBR and TB-2+BBR treatments, respectively, indicating a non-negligible amount of BBR metabolite in the plasma after repeated administration. Only TB-2 was detected in systemic plasma after oral administration of TB-2 and TB-2+BBR in GK rats. Compared with TB-2 treatments alone, there were no significant improvement on the *C*_max_ or AUC of TB-2 (*P* > 0.05) on TB-2+BBR treatments.

**Table 1:** The PK parameters of BBR, TB-2, and M1 in the systemic plasma after oral administration of BBR (66.7 mg/kg), TB-2 (33.3 mg/kg), and their combination in GK rats on day 35 (mean ± SE, *n* = 8).

PK parameters	BBR		M1		TB-2	
	BBR	BBR+TB-2	BBR	BBR+TB-2	TB-2	BBR+TB-2
*T*_1/2_ (h)	28.1 ± 11.7	9.3 ± 2.5	100.1 ± 92.2	23.9 ± 7.4	14.3 ± 5.3	8.6 ± 1.8
*T*_max_ (h)	4.6 ± 1.5	0.9 ± 0.1^∗^	10.4 ± 3.1	9.4 ± 1.2	2.1 ± 0.6	1.1 ± 0.3
*C*_max_ (ng/ml)	5.3 ± 0.4	42.2 ± 17.4^∗^	4.3 ± 1.7	6.6 ± 2.0	18.8 ± 5.4	47.4 ± 14.5
AUC (h ^∗^ ng/ml)	74.8 ± 3.5	154.8 ± 59.7^∗^	56.3 ± 18.5	94.9 ± 26.4	77.5 ± 13.6	100.4 ± 26.2

*The significance of PK parameter was assessed with Welch’s unequal variances t-test. The significance of PK parameters was assessed using two-tailed Student’s t-tests. ^∗^P < 0.05, ^∗∗^P < 0.01, ^∗∗∗^P < 0.001.*

### PK Before and After Hepatic Clearance in Wistar Rats

**FIGURE 4:**
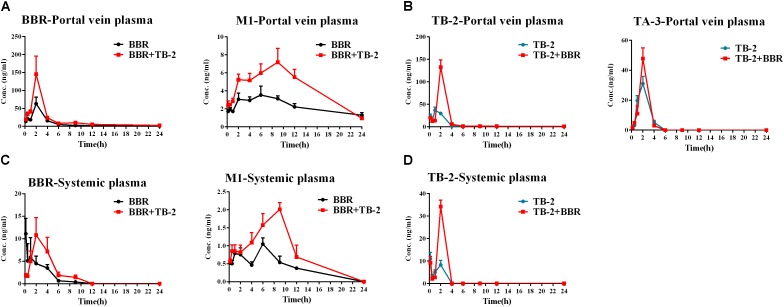
Time–concentration curves of the parent compounds and metabolites after single oral administration of **(A)** BBR (66.7 mg/kg) vs. TB-2+BBR (33.3+66.7 mg/kg), **(B)** TB-2 (33.3 mg/kg) vs. TB-2+BBR (33.3+66.7 mg/kg) in the portal vein plasma, **(C)** BBR (66.7 mg/kg) vs. TB-2+BBR (33.3+66.7 mg/kg), and **(D)** TB-2 (33.3 mg/kg) vs. TB-2+BBR (33.3+66.7 mg/kg) in the systemic plasma of Wistar rats (*n* = 5).

As shown in Figure 4 and Table 2, BBR and M1 were both observed in the portal vein plasma (before hepatic clearance) and systemic plasma (after hepatic clearance), and the presence of TB-2 caused increased levels of BBR and M1 following the oral administration of BBR (66.7 mg/kg) and TB-2+BBR (33.3+66.7 mg/kg) in Wistar rats. Compared with BBR treatments alone, co-administration of TB-2 increased the AUC of BBR by 114% (*P* < 0.01) in the portal vein plasma. In the systemic plasma, the presence of TB-2 only prolonged the *T*_max_ of BBR from 0.167 to 2 h, but it didn’t increase the AUC of BBR with statistical significance (*P* > 0.05). The AUCs of BBR were 6.9- and 9.1-fold higher in the portal vein plasma than those in the systemic plasma on BBR and TB-2+BBR treatments, corresponding to the ER_liver_ of BBR at 86.8 and 90.1%, respectively, indicating that BBR underwent very serious liver first-pass effect.

**Table 2:** The PK parameters of BBR, TB-2, and their corresponding metabolites in the portal vein plasma and systemic plasma after oral administration of BBR (66.7 mg/kg), TB-2 (33.3 mg/kg), and their combination (100 mg/kg) in Wistar rats (*n* = 5).

Portal vein plasma								
PK parameters	BBR		M1		TB-2		TA-3	
	BBR	BBR+TB-2	BBR	BBR+TB-2	TB-2	BBR+TB-2	TB-2	BBR+TB-2
*T*_1/2_ (h)	9.8	10.5	9.7	7	8.5	22.8	0.8	0.5
*T*_max_ (h)	2	2	6	9	1.0	2.0	2.0	2.0
*C*_max_ ± SE (ng/ml)	63.1 ± 18.5	145.3 ± 50.3	3.5 ± 1.0	7.2 ± 1.5^∗^	37.1 ± 6.5	132.6 ± 16.4^∗∗∗^	31.1 ± 4.8	47.7 ± 7.2
AUC ± SE (h ^∗^ ng/ml)	198.0 ± 22.2	424.4 ± 60.5^∗∗^	55.7 ± 3.5	105.5 ± 6.2^∗∗∗^	105.7 ± 7.6	254.8 ± 21.4^∗∗∗^	73.9 ± 5.2	88.3 ± 9.6

**Systemic plasma**								
**PK parameters**	**BBR**		**M1**		**TB-2**			
	**BBR**	**BBR+TB-2**	**BBR**	**BBR+TB-2**	**TB-2**	**BBR+TB-2**		

*T*_1/2_ (h)	1.9	2.3	5.8	1.9	12.8.	23.2		
*T*_max_ (h)	0.2	2	6.0	9.0	0.2	2.0		
*C*_max_ ± SE (ng/ml)	11.1 ± 3.4	10.8 ± 3.9	1.0 ± 0.2	2.0 ± 0.2^∗∗^	11.0 ± 2.9	34.2 ± 3.0^∗∗∗^		
AUC ± SE (h ^∗^ ng/g)	25.1 ± 4.1	42.1 ± 11.7	10.1 ± 0.6	19.7 ± 2.9^∗^	20.3 ± 2.9	56.5 ± 4.6^∗∗∗^		
ER_liver_	87.3%	90.0%	81.9%	81.3%	80.6%	77.8%		

*The calculation of PK parameters used “sparse” method in WinNonlin software (Pharsight 6.2, Raleigh, NC, United States). The significance of PK parameters was assessed with Welch’s unequal variances t-test. ^∗^P < 0.05, ^∗∗^P < 0.01, ^∗∗∗^P < 0.001.*

Concomitantly, in the portal vein plasma, the AUC and *C*_max_ of M1 were increased by 89% (*P* < 0.001) and 105% (*P* < 0.05), respectively. In the systemic plasma, the AUC and *C*_max_ of M1 were similarly enhanced by 95% (*P* < 0.05) and 54% (*P* < 0.05), respectively. Compared with that in the systemic plasma, the AUC of M1 was approximately fivefold higher in the portal vein plasma, corresponding to the ER_liver_ of M1 at 81.9 and 81.3% on BBR and TB-2+BBR treatments, respectively. The AUC ratios between M1 and BBR were 28.1 and 24.9% in the portal vein plasma, and 40.2 and 46.8% in the systemic plasma on BBR and TB-2+BBR treatments, respectively.

Timosaponin B2 and its metabolite TA-3 were observed in the portal vein plasma, whereas only TB-2 was detected in the systemic plasma following oral administration of TB-2 (33.3 mg/kg) and TB-2+BBR (33.3+66.7 mg/kg) in the Wistar rats. In the portal vein plasma, the presence of BBR increased the AUC and *C*_max_ of TB-2 by 257% (*P* < 0.001) and 141% (*P* < 0.001), respectively, along with prolonging the *T*_max_ and *T*_1/2_ of TB-2. Likewise, in the systemic plasma, co-administration of BBR also altered the PK parameters of TB-2, including increasing *C*_max_ by 210% (*P* < 0.001) and AUC by 178% (*P* < 0.001), as well as prolonging *T*_max_ from 0.2 to 2 h and *T*_1/2_ from 12.8 to 23.2 h. However, co-administration of BBR had a negligible effect on the PK behavior of TA-3 in portal vein plasma. Compared with those in the systemic plasma, the AUCs of TB-2 in the portal vein plasma were 4.2- and 3.5-fold higher on TB-2 and TB-2+BBR treatments, respectively, in response to the ER_liver_ of TB-2 at 80.6 and 77.8%, respectively. Due to the increased TB-2 levels and limited change in TA-3 levels, the presence of BBR caused the AUC ratio between TA-3 and TB-2 to decrease from 71% to 35% in the portal vein plasma.

### Liver Distribution in Wistar Rats

**FIGURE 5:**
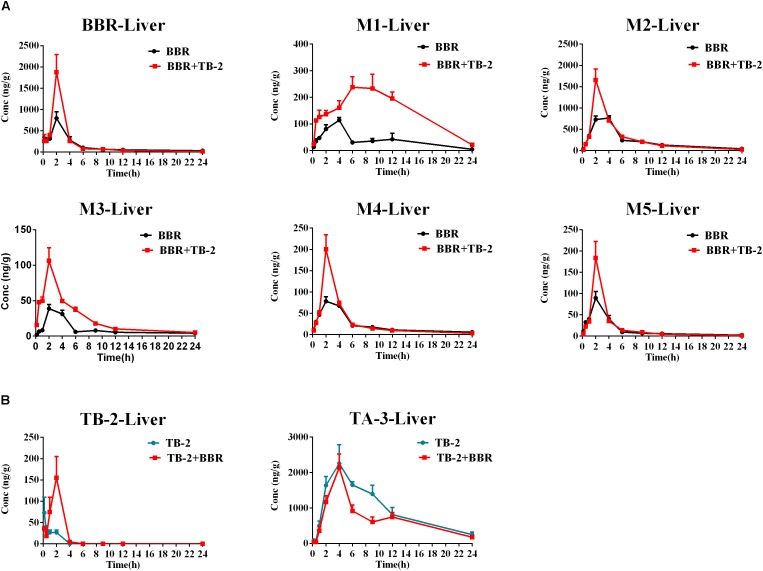
Comparison of time–concentration curves of the parent drugs and metabolites in the liver of Wistar rats after the oral administration of **(A)** BBR (66.7 mg/kg) vs. TB-2+BBR (33.3+66.7 mg/kg) and **(B)** TB-2 (33.3 mg/kg) vs. TB-2+BBR (33.3+66.7 mg/kg) (*n* = 5).

As shown in Figure 5 and Table 3, BBR and its five metabolites, M1–M5, all showed considerable levels in the liver in the BBR- and TB-2+BBR-treated groups. Co-administration of TB-2 improved the *C*_max_ of BBR by 136% (*P* < 0.05), while the increased AUC of BBR didn’t elicit statistical significance. Compared to BBR treatments alone, the *C*_max_ values of BBR metabolites were significantly increased by one- to twofold in the presence of TB-2: 109% for M1 (*P* < 0.05), 117% for M2 (*P* < 0.01), 174% for M3 (*P* < 0.01), 156% for M4 (*P* < 0.01). The corresponding AUCs of BBR metabolites were also significantly improved: 282% for M1 (*P* < 0.001), 144% for M3 (*P* < 0.001), 31% for M4 (*P* < 0.05), and 35% for M5 (*P* < 0.05). These results indicated that co-administration of TB-2 had the greatest impact on the hepatic collection of M1.On TB-2 treatments, the total exposure of BBR metabolites was 246% of that of BBR. This value was mainly attributed to the contribution of M2, which accounted for 182% of the exposure of BBR, along with minor contributions from M1 (29%), M3 (7%), M4 (16%), and M5 (11%). On TB-2+BBR treatments, the total exposure of BBR metabolites was 260% of that of the parent drug, and M2 made the largest contribution (144%), followed by M1 (77%), M3 (12%), M4 (15%), and M5 (11%). In addition, the presence of TB-2 had little effect on the AUC ratio between BBR and its metabolites in the liver, except for M1. The concentration of most metabolites peaked at 2 h, consistent with that of BBR, except those of M1 and M2.

**Table 3:** The PK parameters of BBR, TB-2, and their corresponding metabolites in the liver after oral administration of BBR (66.7 mg/kg), TB-2 (33.3 mg/kg), and their combination in Wistar rats (*n* = 5).

PK parameters	BBR		M1		M2		M3	
	BBR	BBR+TB-2	BBR	BBR+TB-2	BBR	BBR+TB-2	BBR	BBR+TB-2
*T*_1/2_ (h)	14.8	6.9	4.6	4.2	6.8	5.0	18.5	6.2
*T*_max_ (h)	2	2	4.0	6.0	4.0	2	2.0	2.0
*C*_max_ ± SE (ng/g)	794.7 ± 156.8	1876.0 ± 417.6^∗^	114.0 ± 10.6	238.0 ± 39.5^∗^	766.0 ± 54.3	1659.0 ± 254.9^∗∗^	38.8 ± 5.6	106.2 ± 18.2^∗∗^
AUC ± SE (h ^∗^ ng/g)	3255.1 ± 247.5	4600.3 ± 645.1	932.5 ± 155.4	3565.3 ± 256.7^∗∗∗^	5935.8 ± 164.9	6651.2 ± 469.0	234.5 ± 29.3	572.9 ± 34.7^∗∗∗^

**PK parameters**	**M4**		**M5**		**TB-2**		**TA-3**	
	**BBR**	**BBR+TB-2**	**BBR**	**BBR+TB-2**	**TB-2**	**BBR+TB-2**	**TB-2**	**BBR+TB-2**

*T*_1/2_ (h)	9.7	7.0	9.5	6.0	28.3	0.4	6.4	7.6
*T*_max_ (h)	2.0	2.0	2.0	2.0	0.2	2.0	4.0	4.0
*C*_max_ ± SE (ng/g)	78.2 ± 10.7	200.5 ± 33.7^∗∗^	89.4 ± 15.6	183.8 ± 38.4	73.1 ± 36.4	151.2 ± 35.7	2255.2 ± 531.4	2140.2 ± 380.7
AUC ± SE (h^∗^ng/g)	527.5 ± 25.0	690.8 ± 59.6^∗^	362.8 ± 18.7	489.2 ± 59.2^∗^	89.4 ± 19.7	295.0 ± 59.2^∗^	23302.9 ± 3364.2	17099.4 ± 885.5

*The calculation of PK parameters used “sparse” method in WinNonlin software (Pharsight 6.2, NC, United States). The significance of PK parameter was assessed with Welch’s unequal variances t-test. ^∗^P < 0.05, ^∗∗^P < 0.01, ^∗∗∗^P < 0.001.*

Similar to the phenomenon in the portal vein plasma, TB-2 and TA-3 were found in the liver in the TB-2- and TB-2+BBR-treated groups. In the presence of BBR, the AUC of TB-2 were significantly increased by 230% (*P* < 0.05%) with prolonged *T*_max_ and shortened *T*_1/2_. Similar to those in the portal vein plasma, the PK parameters of TA-3 were minimally affected by co-administration with BBR. Moreover, compared with the values in the portal vein plasma, TA-3 showed considerably higher accumulation than TB-2 in the liver, with 259- and 57-fold higher levels than TB-2 in the absence and presence of BBR, respectively.

### Hepatic Metabolism in Primary Hepatocytes

**FIGURE 6:**
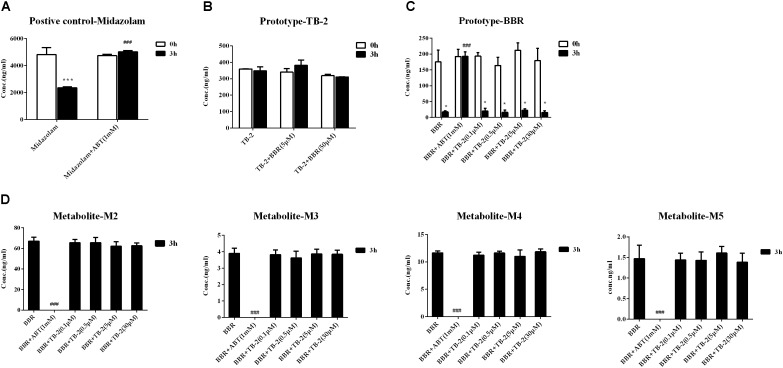
Evaluation of metabolic enzyme-mediated interactions in primary hepatocytes, including **(A)** the metabolism of midazolam (20 μM) in the absence and presence of ABT (1 mM), **(B)** the metabolism of TB-2 in the absence and presence of BBR (5 and 50 μM), **(C)** the metabolism of BBR (1 μM) in the absence and presence of ABT (1 mM) or TB-2 (0.1–30 μM), and **(D)** the generation of M2–M5 in the absence and presence of ABT (1 mM) or TB-2 (0.1–30 μM) before and after incubation for 3 h (*n* = 3). ^∗^*P* < 0.05, ^∗∗^*P* < 0.01, and ^∗∗∗^*P* < 0.001 compared with before incubation; ^#^*P* < 0.05, ^##^*P* < 0.01, and ^###^*P* < 0.001 compared with the absence of inhibitor at 3 h.

As shown in Figure 6A, the positive control midazolam (20 μM) was significantly metabolized after incubation for 3 h in the primary rat hepatocytes, and the presence of ABT, a broad-spectrum CYP enzyme inhibitor, completely abrogated midazolam metabolism. Those indicated the favorable metabolic capacity mediated by the CYP 450 enzyme for evaluating hepatic metabolism in those hepatocytes. However, as presented in Figure 6B, there were no significant changes in the TB-2 levels between before and after incubation in the primary hepatocytes, and the presence of BBR (5 μM, 30 μM) caused no improvement. In contrast, after incubation for 3 h in the primary hepatocytes, BBR underwent significant metabolism, with approximately 80% of BBR disappearing, accompanied by the concomitant appearance of M2–M5, as shown in Figures 6C,D. The presence of ABT completely abrogated BBR metabolism and the generation of M2–M5. In contrast, TB-2 at concentrations ranging from 0.1 to 30 μM did not significantly affect BBR metabolism or the generation of M2–M5. These results indicated that BBR underwent extensive hepatic metabolism, which was limited for TB-2, and neither TB-2 nor BBR had an effect on the hepatic metabolism of each other.

### Intestinal Absorption Transportation in Caco-2 Cells

Transepithelial electrical resistance values were determined before (712 ± 111 Ω/cm^2^) and after (762 ± 109 Ω/cm^2^) transport experiments, with a deviation of <15% from the initial values, confirming that epithelial barrier function was maintained. As displayed in Table 4, an approximately 130-fold greater permeability coefficient of digoxin (5 μM) was observed in the secretory (BL–AP) direction (26.515 ± 1.91 ^∗^ 10^−6^ cm/s) than in the absorptive (AP–BL) direction (0.191 ± 0.018 ^∗^ 10^−6^ cm/s), thus confirming the well-behaved efflux function of Caco-2 cells.

**Table 4:** The apparent permeability (*P*_app_) and efflux ratios (ER_pemeability_) of TB-2 and BBR on single and combination treatments across Caco-2 cells (*n* = 3).

Compounds	Inhibitors	*P*_app_ (AP-BL) (^∗^10^−6^cm/s)	Papp (BL-AP) (^∗^10^−6^ cm/s)	ER_permeability_
Digoxin (10 μM)	–	0.191 ± 0.02	26.515 ± 1.91	138.8
TB-2 (5 μM)	–	0.057 ± 0.032	0.061 ± 0.043	1.1
	BBR (10 μM)	0.081 ± 0.035	0.085 ± 0.035	1
BBR (10 μM)	–	0.038 ± 0.011	2.714 ± 0.355	71.4
	TB-2 (5 μM)	0.048 ± 0.014	1.495 ± 0.499^∗∗^	31.1

*Digoxin was used as a positive control. The significance of *P*_app_ values was assessed using two-tailed Student’s t-tests. ^∗^P < 0.05, ^∗∗^P < 0.01, ^∗∗∗^P < 0.001.*

There were no significant differences in the *P*_app_ values of TB-2 in the absorptive (0.057 ± 0.032 ^∗^ 10^−6^ cm/s) and secretory (0.061 ± 0.043 ^∗^ 10^−6^ cm/s) directions on TB-2 (5 μM) treatments, corresponding to an ER_permeability_ at 1.1. Co-incubation with BBR (10 μM) had no significant effect on the absorptive and secretory permeability of TB-2. In contrast, the *P*_app_ of BBR was markedly higher in the secretory side (2.714 ± 0.355 ^∗^ 10^−6^ cm/s) than that in the absorptive (0.038 ± 0.011 ^∗^ 10^−6^ cm/s) side on BBR treatments, and the ER_permeability_ of BBR was up to 71.4. The presence of TB-2 resulted in a significant reduction in the *P*_app_ (BL–AP) from 2.714 ± 0.355 ^∗^ 10^−6^ to 1.495 ± 0.499 ^∗^ 10^−6^ cm/s, while this reduction was limited for *P*_app_ (AP–BL) (0.038 ± 0.011 ^∗^ 10^−6^ cm/s vs. 0.048 ± 0.014 ^∗^ 10^−6^ cm/s). Correspondingly, the ER_permeability_ of BBR was decreased from 71.4 to 31.1 in the presence of TB-2 (5 μM). In brief, TB-2 could significantly inhibit the excretory permeability of BBR, whereas BBR barely affected the limited intestinal absorption of TB-2 in Caco-2 cells.

## Discussion

Based on the long history of clinical usage of *Zhimu*-*Huangbo* to treat “*Xiaoke*” symptom, TB-2 isolated from *Zhimu* and BBR derived from *Huangbo* were used as a combination therapy for diabetes in GK rats, a preferable model to mimic the hyperglycemic state of humans (Akash et al., 2013). Previously reported studies verifying the hypoglycemic efficacy of BBR or TB-2 were conducted in chemically induced diabetic rats (Liu et al., 2008); however, none were performed in GK rats. In the current study, no obvious side effects were observed throughout the whole experiment, indicating the short-term safety of these compounds in GK rats. We found there were no significant improvements on treating diabetes on either BBR (66.7 mg/kg) or TB-2 (33.3 mg/kg) treatments (Figure 2). In contrast, BBR (66.7 mg/kg) co-administered with TB-2 (33.3 mg/kg) achieved significantly anti-diabetic efficacy, as evidenced by the parameters of FBG, NFBG, and OGTT in GK rats, and those parameters were much better than those on single compound treatments (Figure 2).

Berberine is often compared with metformin, the first-line anti-T2D drug (Lee et al., 2006); both are typical AMPK activators to suppress hepatic gluconeogenesis. However, to match the similar anti-diabetic efficacy of metformin, the *in vitro* and *in vivo* studies verified the requirements for the same or higher dosage of BBR (Xu et al., 2014; Liu et al., 2015), likely due to its low bioavailability (<1%) (Liu et al., 2016). However, at oral dosages of BBR over 0.9 g/day, diabetic patients were prone to suffer gastrointestinal side effects (Yin et al., 2008). Therefore, reducing the oral dosage of BBR while sustaining efficacy is of great clinical significance. In our study, when combined with TB-2, only one-third of the oral dosage (66.7 mg/kg) was required for BBR to achieve comparable hypoglycemic efficacy to metformin (200 mg/kg), as evidenced by OGTT and FBG results. Some parameters (NFBG, liver, and kidney coefficients) were even better (Figure 2). These results indicated the superiority of combination treatments for treating diabetes.

**FIGURE 7:**
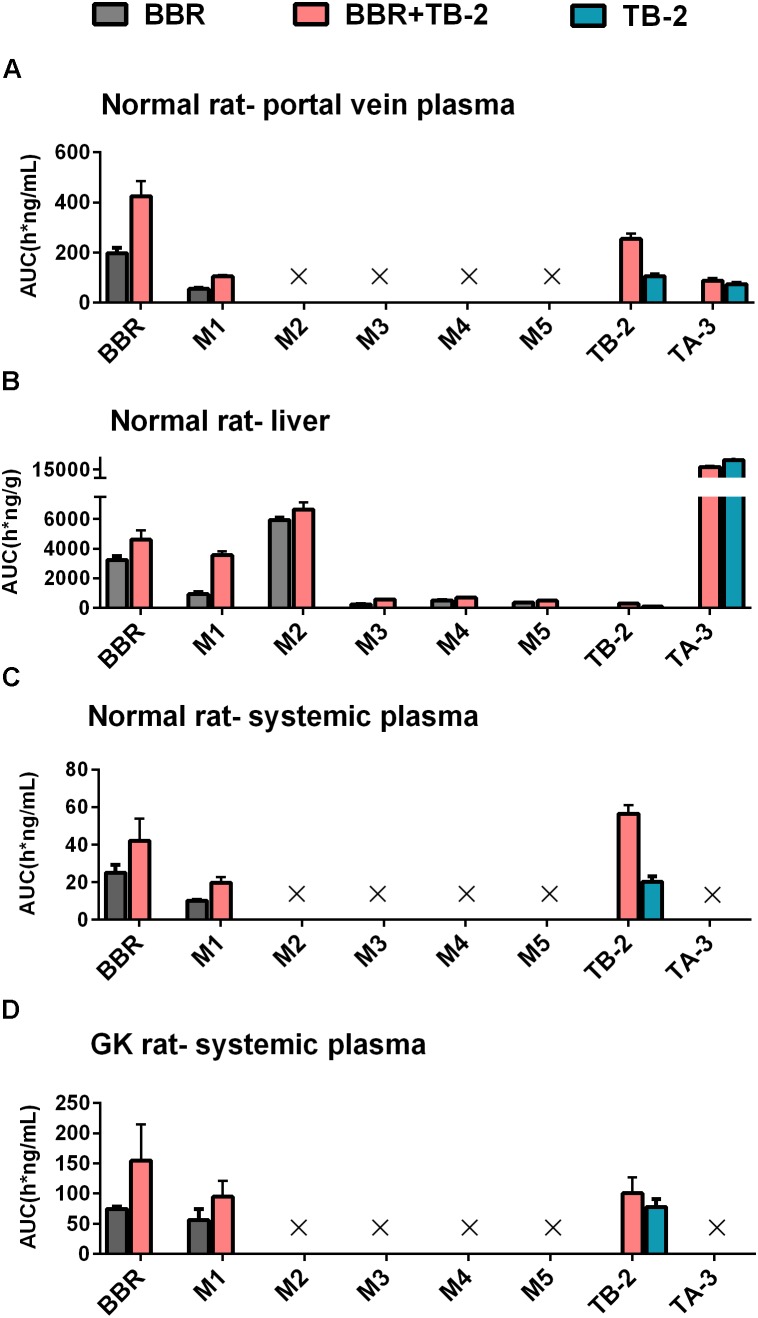
The compared AUCs of TB-2, BBR, and their metabolites in the **(A)** portal vein plasma, **(B)** liver, and **(C)** systemic plasma of normal rats, as well as **(D)** systemic plasma of GK rats, following oral administration of BBR (66.7 mg/kg), TB-2 (33.3 mg/kg), and TB-2+BBR (100 mg/kg).

On the one hand, an enhancement in pharmacology is associated with introducing more effective compounds to cause additive or synergistic effects on combination treatments (Prabhakar et al., 2014). BBR elicited anti-diabetic efficacy through multiple target pathways, such as activating AMPK, inhibiting protein-tyrosine phosphatase 1B (PTP1B), increasing InsR, and inhibiting liver gluconeogenesis (Zhang et al., 2008; Dong et al., 2012; Pirillo and Catapano, 2015). The gut microflora may also play a role in the anti-diabetic efficacy of BBR (Wang Y. et al., 2017). The metabolites of BBR, M1–M5, have all shown some potentials for diabetic management through different pathways, involved of InsR, AMPK and succinate dehydrogenase activation, and glucose-6-phosphatase and hexokinase inhibition (Li et al., 2011; Wang K. et al., 2017; Yang et al., 2017). TB-2 was able to improve insulin sensitivity through the insulin receptor substrate-1/phosphatidylinositol 3-kinase/Akt pathway (Yuan et al., 2016). The hypoglycemic efficacy of TA-3 was likely associated with hepatic gluconeogenesis or glycogenolysis inhibition (Nakashima et al., 1993). Notably, many of the above-mentioned anti-diabetic pathways happened in the liver, such as activating AMPK, increasing InsR expression, and inhibiting liver gluconeogenesis (Lee et al., 2006; Turner et al., 2008; Xia et al., 2011; Yao et al., 2015). Following oral administration of TB-2 or BBR on single or combination treatments, we found that the circulating levels of BBR and TB-2 were both low in rats (Figures 3, 4), in line with the reported poor bioavailability of TB-2 (1.2%) and BBR (<1%) (Cai et al., 2008; Liu et al., 2016). Compared to the poor circulating levels of BBR, the hepatic levels of BBR were two orders of magnitude higher. The discrepancy can be explained by the AUCs of BBR that were approximately 10-fold higher in the portal vein plasma, responsible for transporting compounds to liver, than those in the systemic plasma (Figure 4). Besides, five metabolites of BBR also showed substantial collection in the liver, where their total exposure was approximately 200-fold of circulating levels of BBR (Figure 5). Of note, the AUCs of M1, a metabolite that exhibited more hypoglycemic capacity than BBR (Yang et al., 2017), also showed very high hepatic collection (Figure 7B). Interestingly, the hepatic collection of TA-3, an active metabolite of TB-2, was 259- and 57-fold higher than those of TB-2 on TB-2 and TB-2+BBR treatments, respectively (Figure 7B). In sum, compared with the single compound treatments, the combination of TB-2 and BBR can introduce more compounds, involving the parent drugs and active metabolites *in vivo*, to cause additive or synergistic effects, leading to the enhanced efficacy in GK rats. On the other hand, DDIs can improve the exposure of drugs and active metabolites *in vivo*, especially for the target organs, which also bring about potentiation effects (Prabhakar et al., 2014). In normal rats, co-administration also improved the levels by 41–114% for BBR, 141–230% for TB-2, and 12–282% for BBR metabolites in the plasma and liver (Figure 7). Similarly, the improved circulating levels of BBR were verified after repeated oral administration in GK rats (Figure 7D). Thus, the improved exposure of parent drugs and active metabolites in the plasma and target organ also contributed to the enhanced anti-diabetic efficacy on TB-2+BBR treatments.

Given that pharmacokinetic and metabolic interactions usually occur through intestinal absorption or hepatic metabolism, those influencing factors were evaluated *in vitro*. Caco-2 cells, derived from human colonic cancer cells, are appropriate for studying intestinal absorption *in vitro* due to their ability to mimic intestinal epidermal cell patterns (Proctor et al., 2010). The ER_permeability_ of BBR was as high as 71.4, in agreement with that BBR is a typical substrate of P-gp (Zhang et al., 2011). However, there was no significant efflux of TB-2, with an ER_permeability_ of only 1. This seemed to contradict our previous finding that TB-2 was a substrate of both multidrug resistance-associated protein (BCRP) and multidrug resistance-associated protein 2 (MRP2) (Sheng et al., 2015). As reported, despite the detection of the mRNAs of many intestinal transporters, only P-gp expression in Caco-2 cells was stable and comparable (within twofold) to that in the human intestine (Troutman and Thakker, 2003). In contrast, the expression of MRP-2 and BCRP in Caco-2 cells is unstable and much lower than those in the human intestine, with one study even describing a 100-fold lower expression of BCRP in Caco-2 cells (Taipalensuu et al., 2001; Proctor et al., 2010). Therefore, the low ER_permeability_ of TB-2 was possibly due to the poor BCRP and MRP2 expression in the Caco-2 cells, and TB-2 was very likely a P-gp inhibitor, rather than a P-gp substrate. This can explain why TB-2 significantly decreased the ER_permeability_ of BBR from 71.4 to 31.1, and co-incubation with BBR did not improve TB-2 excretion in Caco-2 cells (Table 4). If the compounds functioned as P-gp inhibitors by interfering with ATP hydrolysis or altering the integrity of cell membrane lipids, an inconsistency between compounds as inhibitors and substrates might appear (Silva et al., 2015). Likewise, BBR was also reported to be a BCRP inhibitor, rather than a substrate (Zhang et al., 2011; Tan et al., 2013), and BBR was neither an inhibitor nor a substrate of MRP2 (Gu et al., 2015; Qian et al., 2016). Overall, the mutual inhibition of TB-2 and BBR, as P-gp and BCRP inhibitors, respectively, hampered the intestinal absorption of each other, in agreement with the improved TB-2 and BBR levels in the portal vein plasma on combination treatments (Figures 4, 5). After incubation with rat primary hepatocytes, BBR underwent extensive CYP 450-mediated metabolism to generate M2–M5, but such metabolism was limited for TB-2. Furthermore, neither TB-2 nor BBR affected the other’s hepatic metabolism. These findings were consistent with the phenomenon that the presence of TB-2 resulted in the increased BBR and M2–M5 levels, but rarely affected the exposure ratio between BBR and M2–M5 in the liver of rats (Table 3). Hence, the improved accumulation of M2–M5 simply resulted from an increased quantity of metabolizable BBR in the liver, rather than DDIs mediated by the CYP 450 enzyme. Unlike M2–M5, the generation of M1 was attributed to microbiota- rather than liver-mediated BBR metabolism (Wang K. et al., 2017), in line with its absence in primary hepatocytes after BBR incubation (Figure 6), considerable exposure in the portal vein plasma (Figure 4A), and longer *T*_max_ than the other BBR metabolites in the liver (Figure 5). Co-administration of TB-2 increased the AUC_M1_/AUC_BBR_ from 28.6% to 77.5% in the liver (Table 3), which was probably correlated with the microbiota-mediated interaction between BBR (or M1) and TB-2 (or TA-3); this awaits further study. Briefly, the DDIs between BBR and TB-2 were probably mediated by intestinal absorption, rather than hepatic metabolism.

This study has some limitations that warrant further examination. For instance, questions still need to be answered regarding whether the co-administration of TB-2 and BBR influences glycosylated hemoglobin levels, insulin, and lipid parameters. Whether gut microbiota plays a role in the enhanced anti-diabetic efficacy on combination treatments, and the long-term safety and diabetic efficacy in other species requires further verification.

The famous Chinese scientist Youyou Tu, a winner of the Nobel Prize for Medicine in 2015 for the discovery of artemisinin inspired by historical records in ancient China, declared that artemisinin was a gift from Traditional Chinese Medicine (TCM) to the world (Shen, 2015). By comparison, the combination of herb medicines, namely, the TCM compatibility, contained more complicated and valuable ancestral wisdom through a long history of clinical trials in China. However, it has not been scientifically explored and utilized, recently. This study represented a primary attempt to develop combined plant ingredients, inspired by historical clinical experience with the compatibility of herb pairs, to treat diabetes. We hope it can motivate more researchers to scientifically explore the TCM compatibility, which may be the next gift from TCM to the world.

## Conclusion

In this study, co-administration of TB-2 (33.3 mg/kg/day) and BBR (66.7 mg/kg/day) displayed significantly enhanced anti-diabetic efficacy in GK rats, which surpassed that of either compound alone, and was comparable to that of metformin (200 mg/kg/day). On the one hand, this enhanced efficacy was associated with the sum of the effective substances with multiple targets *in vivo*, including the active parent compounds (TB-2, BBR) and the relevant metabolites (TA-3 and M1–M5), especially for the substantial exposure of active metabolites and BBR in the target organ, liver. On the other hand, except for TA-3, co-administration improved the levels of those effective substances in the plasma and liver via intestinal absorption, rather than hepatic metabolism, which also played an important role in the potentiation effects on diabetes. Briefly, this study represented a successful attempt to assess the effect of combined plant ingredients on diabetes, inspired by historical clinical experience with herb pairs.

## Ethics Statement

The animal studies were approved by the Institutional Animal Care and Use Committee of the Shanghai Institute of Materia Medica, China Academic Science, which has been described in the manuscript.

## Author Contributions

XT, FL, YL, ZL, GP, and CH conceived and designed the experiments. HL, PH, MC, ZX, LH, and YZ assisted with the experiments. ZS and YZ analyzed the data. XT wrote the paper. YZ, GP, and CH critically revised the manuscript. All the authors read and reviewed the final manuscript.

## Conflict of Interest Statement

The authors declare that the research was conducted in the absence of any commercial or financial relationships that could be construed as a potential conflict of interest.

## References

[B1] Akash M. Rehman K. Chen S. (2013). Goto-kakizaki rats: its suitability as non-obese diabetic animal model for spontaneous type 2 diabetes mellitus. *Curr. Diabetes Rev.* 9 387–396. 10.2174/15733998113099990069 23855509

[B2] Cai F. Sun L. N. Gao S. H. Yang Y. Yang Q. Chen W. S. (2008). A rapid and sensitive liquid chromatography-tandem mass spectrometric method for the determination of timosaponin B-II in blood plasma and a study of the pharmacokinetics of saponin in the rat. *J. Pharm. Biomed. Anal.* 48 1411–1416. 10.1016/j.jpba.2008.09.032 19027255

[B3] Chen C. Wu Z. T. Ma L. L. Ni X. Lin Y. F. Wang L. (2015). Organic anion-transporting polypeptides contribute to the hepatic uptake of berberine. *Xenobiotica* 45 1138–1146. 10.3109/00498254.2015.1042537 26068524

[B4] Dong H. Wang N. Zhao L. Lu F. (2012). Berberine in the treatment of type 2 diabetes mellitus: a systemic review and meta-analysis. *Evid. Based Complement. Alternat. Med.* 2012:591654. 10.1155/2012/591654 23118793 PMC3478874

[B5] Fu Z. W. Li Z. X. Xue R. Jiang J. Huang C. G. (2015). Stereoisomerism metabolites found in rats after oral administration of timosaponin B-II using HPLC-Q-TOF-MS and NMR methods. *RSC Adv.* 5 60650–60657. 10.1039/C5RA09133K

[B6] Gu Q. Shao Y. An R. Wang Y. Zhang Y. Wang X. (2015). Influence of active ingredients in gegen qinlian decoction on uptake and transport of baicalin in Caco-2 cell model. *Zhongchengyao* 37 694–699.

[B7] Guariguata L. Whiting D. R. Hambleton I. Beagley J. Linnenkamp U. Shaw J. E. (2014). Global estimates of diabetes prevalence for 2013 and projections for 2035. *Diabetes Res. Clin. Pract.* 103 137–149. 10.1016/j.diabres.2013.11.002 24630390

[B8] Jeong H. Kwon H. J. Kim M. K. (2009). Hypoglycemic effect of *Chlorella vulgaris* intake in type 2 diabetic Goto-Kakizaki and normal Wistar rats. *Nutr. Res. Pract.* 3 23–30. 10.4162/nrp.2009.3.1.23 20016698 PMC2788164

[B9] Jia Y. Fu Z. W. Li Z. X. Hu P. Xue R. Chen M. C. (2015). In-vivo and in-vitro metabolism study of timosaponin B-II using HPLC-ESI-MS (n). *Chromatographia* 78 1175–1184. 10.1007/s10337-015-2927-6

[B10] Lee Y. S. Kim W. S. Kim K. H. Yoon M. J. Cho H. J. Shen Y. (2006). Berberine, a natural plant product, activates AMP-activated protein kinase with beneficial metabolic effects in diabetic and insulin-resistant states. *Diabetes* 55 2256–2264. 10.2337/db06-0006 16873688

[B11] Li Y. Ren G. Wang Y. X. Kong W. J. Yang P. Wang Y. M. (2011). Bioactivities of berberine metabolites after transformation through CYP450 isoenzymes. *J. Transl. Med.* 9:62. 10.1186/1479-5876-9-62 21569619 PMC3103436

[B12] Liu C. Wang Z. Song Y. Wu D. Zheng X. Li P. (2015). Effects of berberine on amelioration of hyperglycemia and oxidative stress in high glucose and high fat diet-induced diabetic hamsters in vivo. *Biomed. Res. Int.* 2015:313808. 10.1155/2015/313808 25705654 PMC4331319

[B13] Liu C. S. Zheng Y. R. Zhang Y. F. Long X. Y. (2016). Research progress on berberine with a special focus on its oral bioavailability. *Fitoterapia* 109 274–282. 10.1016/j.fitote.2016.02.001 26851175

[B14] Liu W. Liu P. Tao S. Deng Y. Li X. Lan T. (2008). Berberine inhibits aldose reductase and oxidative stress in rat mesangial cells cultured under high glucose. *Arch. Biochem. Biophys.* 475 128–134. 10.1016/j.abb.2008.04.022 18471986

[B15] Ma C. Fan M. Tang Y. Li Z. Sun Z. Ye G. (2008). Identification of major alkaloids and steroidal saponins in rat serum by HPLC-diode array detection-MS/MS following oral administration of Huangbai-Zhimu herb-pair Extract. *Biomed. Chromatogr.* 22 835–850. 10.1002/bmc.1000 18318017

[B16] Ma C. H. Li Z. X. Wang L. X. Tang Y. H. Xiao H. B. Huang C. G. (2009). Identification of major alkaloids in rat urine by HPLC/DAD/ESI-MS/MS method following oral administration of cortex phellodendri decoction. *Helv. Chim. Acta* 92 379–398. 10.1002/hlca.200800315

[B17] Nakashima N. Kimura I. Kimura M. Matsuura H. (1993). Isolation of pseudoprototimosaponin AIII from rhizomes of *Anemarrhena asphodeloides* and its hypoglycemic activity in streptozotocin-induced diabetic mice. *J. Nat. Prod.* 56 345–350. 10.1021/np50093a006 8482946

[B18] Nathan D. M. (2007). Finding new treatments for diabetes–how many, how fast... how good? *N. Engl. J. Med.* 356 437–440. 10.1056/NEJMp068294 17267901

[B19] Nathan D. M. Buse J. B. Davidson M. B. Ferrannini E. Holman R. R. Sherwin R. (2009). Medical management of hyperglycemia in type 2 diabetes: a consensus algorithm for the initiation and adjustment of therapy: a consensus statement of the American diabetes association and the European association for the study of diabetes. *Diabetes Care* 32 193–203. 10.2337/dc08-9025 18945920 PMC2606813

[B20] Pirillo A. Catapano A. L. (2015). Berberine, a plant alkaloid with lipid- and glucose-lowering properties: from in vitro evidence to clinical studies. *Atherosclerosis* 243 449–461. 10.1016/j.atherosclerosis.2015.09.032 26520899

[B21] Prabhakar P. K. Kumar A. Doble M. (2014). Combination therapy: a new strategy to manage diabetes and its complications. *Phytomedicine* 21 123–130. 10.1016/j.phymed.2013.08.020 24074610

[B22] Proctor W. R. Ming X. Thakker D. R. (2010). “In vitro techniques to study drug-drug interactions involving transport: Caco-2 model for study of P-glycoprotein and other transporters,” in *Enzyme- and Transporter-Based Drug-Drug Interactions: Progress and Future Challenges*, eds Pang K. S. Rodrigues A. D. Peter R. M. (New York, NY: Springer), 257–282.

[B23] Qian Z. Huang C. Shen C. Meng X. Chen Z. Hu T. (2016). The permeability characteristics and interaction of the main components from Zhizi Bopi decoction in the MDCK cell model. *Xenobiotica* 46 733–742. 10.3109/00498254.2015.1113575 26634613

[B24] Shen B. (2015). A new golden age of natural products drug discovery. *Cell* 163 1297–1300. 10.1016/j.cell.2015.11.031 26638061 PMC5070666

[B25] Shen L. Hillebrand A. Wang D. Q. Liu M. (2012). Isolation and primary culture of rat hepatic cells. *J. Vis. Exp.* 64:3917. 10.3791/3917 22781923 PMC3471302

[B26] Sheng J. Tian X. Xu G. Wu Z. Chen C. Wang L. (2015). The hepatobiliary disposition of timosaponin b2 is highly dependent on influx/efflux transporters but not metabolism. *Drug Metab. Dispos.* 43 63–72. 10.1124/dmd.114.059923 25336752

[B27] Silva R. Vilas-Boas V. Carmo H. Dinis-Oliveira R. J. Carvalho F. De Lourdes Bastos M. (2015). Modulation of P-glycoprotein efflux pump: induction and activation as a therapeutic strategy. *Pharmacol. Ther.* 149 1–123. 10.1016/j.pharmthera.2014.11.013 25435018

[B28] Song L. Liu H. Wang Y. Wang Y. Liu J. Zhou Z. (2015). Application of GC/MS-based metabonomic profiling in studying the therapeutic effects of Huangbai-Zhimu herb-pair (HZ) extract on streptozotocin-induced type 2 diabetes in mice. *J. Chromatogr. B Analyt. Technol. Biomed. Life Sci.* 997 96–104. 10.1016/j.jchromb.2015.05.003 26094210

[B29] Taipalensuu J. Tornblom H. Lindberg G. Einarsson C. Sjoqvist F. Melhus H. (2001). Correlation of gene expression of ten drug efflux proteins of the ATP-binding cassette transporter family in normal human jejunum and in human intestinal epithelial Caco-2 cell monolayers. *J. Pharmacol. Exp. Ther.* 299 164–170. 11561076

[B30] Tan K. W. Li Y. Paxton J. W. Birch N. P. Scheepens A. (2013). Identification of novel dietary phytochemicals inhibiting the efflux transporter breast cancer resistance protein (BCRP/ABCG2). *Food Chem.* 138 2267–2274. 10.1016/j.foodchem.2012.12.021 23497885

[B31] Tang Y. H. Sun Z. L. Fan M. S. Li Z. X. Huang C. G. (2012). Anti-diabetic effects of TongGuanWan, a Chinese traditional herbal formula, in C57BL/KsJ-db/db mice. *Planta Med.* 78 18–23. 10.1055/s-0031-1280268 22002851

[B32] Tian X. Gao Y. Xu Z. Lian S. Ma Y. Guo X. (2016a). Pharmacokinetics of mangiferin and its metabolite-Norathyriol, Part 1: systemic evaluation of hepatic first-pass effect in vitro and in vivo. *Biofactors* 42 533–544. 10.1002/biof.1291 27130074

[B33] Tian X. Xu Z. Li Z. Ma Y. Lian S. Guo X. (2016b). Pharmacokinetics of mangiferin and its metabolite-norathyriol, Part 2: influence of UGT, CYP450, P-gp, and enterobacteria and the potential interaction in Rhizoma Anemarrhenae decoction with timosaponin B2 as the major contributor. *Biofactors* 42 545–555. 10.1002/biof.1290 27151461

[B34] Tian X. Zhang Y. Li Z. Hu P. Chen M. Sun Z. (2016c). Systematic and comprehensive strategy for metabolite profiling in bioanalysis using software-assisted HPLC-Q-TOF: magnoflorine as an example. *Anal. Bioanal. Chem.* 408 2239–2254. 10.1007/s00216-015-9254-5 26873213

[B35] Tian X. Li Z. Lin Y. Chen M. Pan G. Huang C. (2014). Study on the PK profiles of magnoflorine and its potential interaction in *Cortex phellodendri* decoction by LC-MS/MS. *Anal. Bioanal. Chem.* 406 841–849. 10.1007/s00216-013-7530-9 24337185

[B36] Troutman M. D. Thakker D. R. (2003). Novel experimental parameters to quantify the modulation of absorptive and secretory transport of compounds by P-glycoprotein in cell culture models of intestinal epithelium. *Pharm. Res.* 20 1210–1224. 10.1023/A:1025001131513 12948019

[B37] Turner N. Li J.-Y. Gosby A. To S. W. C. Cheng Z. Miyoshi H. (2008). Berberine and its more biologically available derivative, dihydroberberine, inhibit mitochondrial respiratory complex I: a mechanism for the action of berberine to activate AMP-activated protein kinase and improve insulin action. *Diabetes* 57 1414–1418. 10.2337/db07-1552 18285556

[B38] Wang K. Feng X. Chai L. Cao S. Qiu F. (2017). The metabolism of berberine and its contribution to the pharmacological effects. *Drug Metab. Rev.* 49 139–157. 10.1080/03602532.2017.1306544 28290706

[B39] Wang Y. Shou J. W. Li X. Y. Zhao Z. X. Fu J. He C. Y. (2017). Berberine-induced bioactive metabolites of the gut microbiota improve energy metabolism. *Metabolism* 70 72–84. 10.1016/j.metabol.2017.02.003 28403947

[B40] Wang Q. (2003). The present situation of TCM treatment for diabetes and its researches. *J. Tradit. Chin. Med.* 23 67–73. 12747208

[B41] Xia X. Yan J. Shen Y. Tang K. Yin J. Zhang Y. (2011). Berberine improves glucose metabolism in diabetic rats by inhibition of hepatic gluconeogenesis. *PLoS One* 6:e16556. 10.1371/journal.pone.0016556 21304897 PMC3033390

[B42] Xu M. Xiao Y. Yin J. Hou W. Yu X. Shen L. (2014). Berberine promotes glucose consumption independently of AMP-activated protein kinase activation. *PLoS One* 9:e103702. 10.1371/journal.pone.0103702 25072399 PMC4114874

[B43] Yang N. Sun R. B. Chen X. L. Zhen L. Ge C. Zhao Y. Q. (2017). In vitro assessment of the glucose-lowering effects of berberrubine-9-O-beta-D-glucuronide, an active metabolite of berberrubine. *Acta Pharmacol. Sin.* 38 351–361. 10.1038/aps.2016.120 28042874 PMC5342660

[B44] Yao J. Kong W. Jiang J. (2015). Learning from berberine: treating chronic diseases through multiple targets. *Sci. China Life Sci.* 58 854–859. 10.1007/s11427-013-4568-z 24174332

[B45] Yin J. Xing H. Ye J. (2008). Efficacy of berberine in patients with type 2 diabetes mellitus. *Metabolism* 57 712–717. 10.1016/j.metabol.2008.01.013 18442638 PMC2410097

[B46] Yuan Y. L. Guo C. R. Cui L. L. Ruan S. X. Zhang C. F. Ji D. (2015). Timosaponin B-II ameliorates diabetic nephropathy via TXNIP, mTOR, and NF-kappaB signaling pathways in alloxan-induced mice. *Drug Des. Devel. Ther.* 9 6247–6258. 10.2147/DDDT.S96435 26664046 PMC4669930

[B47] Yuan Y. L. Lin B. Q. Zhang C. F. Cui L. L. Ruan S. X. Yang Z. L. (2016). Timosaponin B-II ameliorates palmitate-induced insulin resistance and inflammation via IRS-1/PI3K/Akt and IKK/NF-[Formula: see text]B pathways. *Am. J. Chin. Med.* 44 755–769. 10.1142/S0192415X16500415 27222060

[B48] Zhang J. Zhuang P. Wang Y. Song L. Zhang M. Lu Z. (2014). Reversal of muscle atrophy by Zhimu-Huangbai herb-pair via Akt/mTOR/FoxO3 signal pathway in streptozotocin-induced diabetic mice. *PLoS One* 9:e100918. 10.1371/journal.pone.0100918 24968071 PMC4072704

[B49] Zhang M. Chen L. (2012). Berberine in type 2 diabetes therapy: a new perspective for an old antidiarrheal drug? *Acta Pharm. Sin. B* 2 379–386. 10.1016/j.apsb.2012.06.004

[B50] Zhang X. Qiu F. Jiang J. Gao C. Tan Y. (2011). Intestinal absorption mechanisms of berberine, palmatine, jateorhizine, and coptisine: involvement of P-glycoprotein. *Xenobiotica* 41 290–296. 10.3109/00498254.2010.529180 21319959

[B51] Zhang Y. Li X. Zou D. Liu W. Yang J. Zhu N. (2008). Treatment of type 2 diabetes and dyslipidemia with the natural plant alkaloid berberine. *J. Clin. Endocrinol. Metab.* 93 2559–2565. 10.1210/jc.2007-2404 18397984

[B52] Zhao L. C. Gao H. C. Zhao Y. X. Lin D. H. (2012). Metabonomic analysis of the therapeutic effect of Zhibai Dihuang Pill in treatment of streptozotocin-induced diabetic nephropathy. *J. Ethnopharmacol.* 142 647–656. 10.1016/j.jep.2012.05.031 22687255

